# Supplementary material for: Genome-wide analysis of mRNA regionalization in a giant single cell
Source: Proc Natl Acad Sci U S A. 2026 Jun 15;123(25):e2537760123. doi: 10.1073/pnas.2537760123 (PMC13291615; doi:10.1073/pnas.2537760123)
Supplement: Supplementary file 1 — Appendix 01 (PDF) [file pnas.2537760123.sapp.pdf]

PNAS

Supporting Information for

Genome-wide analysis of mRNA regionalization in a giant single cell

Ashley R. Albright, Connie Yan, David Angeles-Albores, Yina Hudnall, Tatyana Makushok, Jamarc Allen-Henderson, Wallace F. Marshall

Wallace Marshall
Email: Wallace.Marshall@ucsf.edu

This PDF file includes:

Figures S1 to S3
Legends for Supplementary Files S1 to S10

Figures

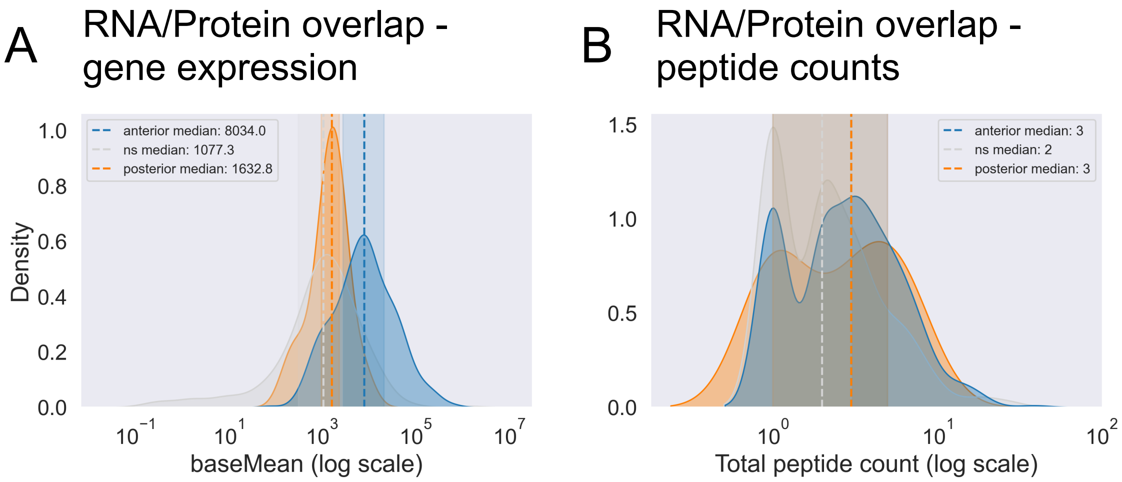

Supplementary Figure 1. Transcript and protein abundance of transcripts detected in oral apparatus mass spectrometry. (A) Kernel density estimate of mean normalized counts for transcripts with baseMean > 0 and corresponding proteins detected in Wei et al. 2020 [13] (n: anterior = 440, ns = 335, posterior = 13). (B) Total peptide counts from Wei et al 2020 [13] with corresponding transcripts in panel (A). (A) and (B) are colored according to the identity as determined by our analysis: anterior-enriched (blue), posterior-enriched (orange), and non-significant (gray). Dashed vertical lines indicate group medians; shaded bands indicate interquartile ranges.

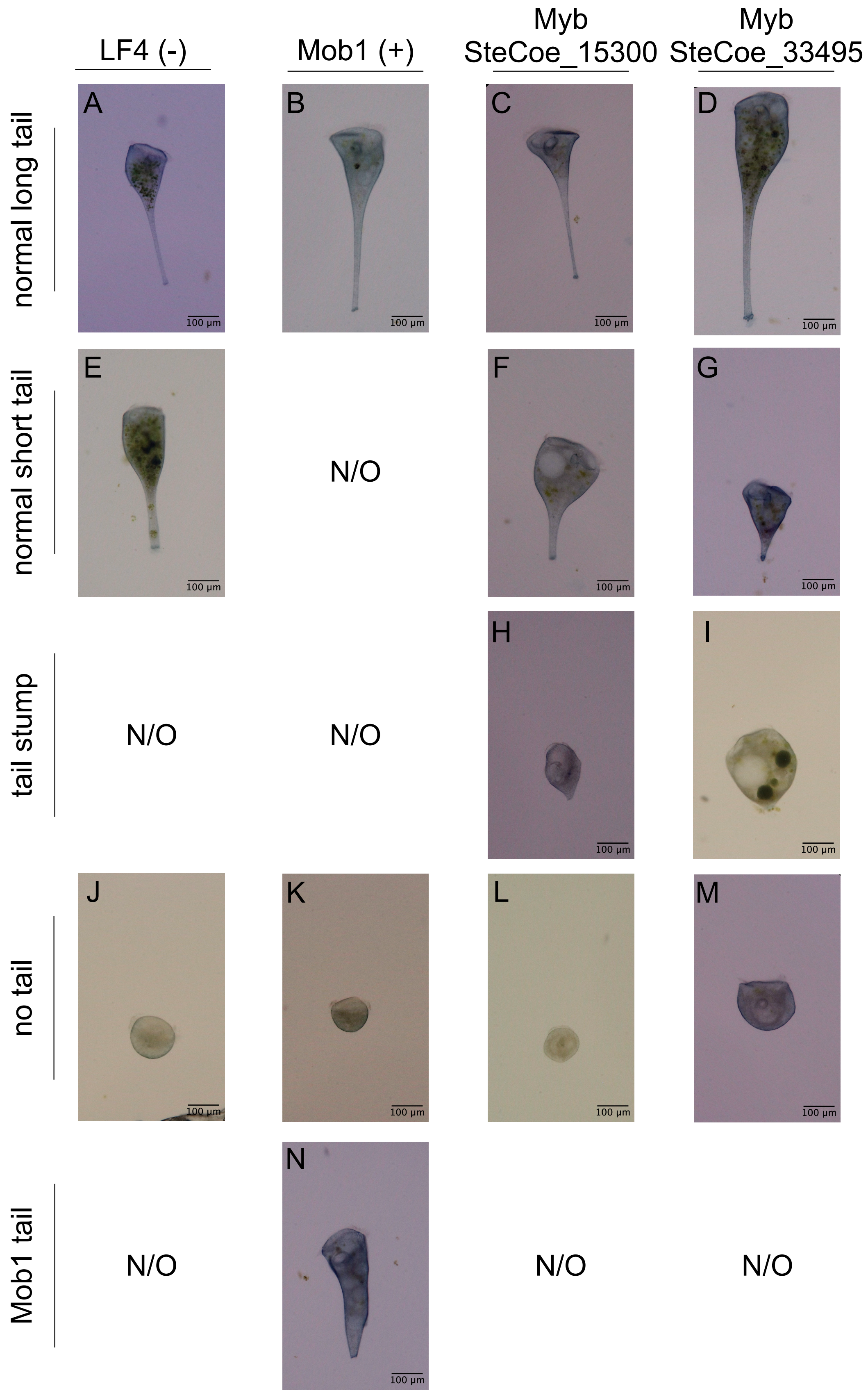
LF4 (-)
Mob1 (+)
Myb SteCoe_15300
Myb SteCoe_33495
normal long tail
normal short tail
tail stump
no tail
Mob1 tail
A
B
C
D
E
N/O
F
G
N/O
N/O
H
I
J
K
L
M
N/O
N
N/O
N/O
100 μm

Supplementary Figure 2. Representative phenotypes from control and RNAi knockdown conditions in anterior half-cells. Brightfield images of anterior halves of knockdown cells 24 hours post-bisection. Normal long tails with tapered shape and holdfast appeared in all conditions (A-D). Normal short tails with a tapered shape and holdfast appeared in (E) LF4, (F) SteCoe_15300, and (G) SteCoe_33495. Appearance of a posterior stump with a holdfast but no tapered region, which was counted as no tail in our analysis, only appeared in MYB knockdown cells (H) SteCoe_15300 and (I) SteCoe_33495. Cells with no visible tail occurred in all conditions at least once (J-M). The characteristic Mob1 phenotype characterized by a thick cylindrical body shape was only seen in (N) Mob1 knockdown cells. N/O = not observed.

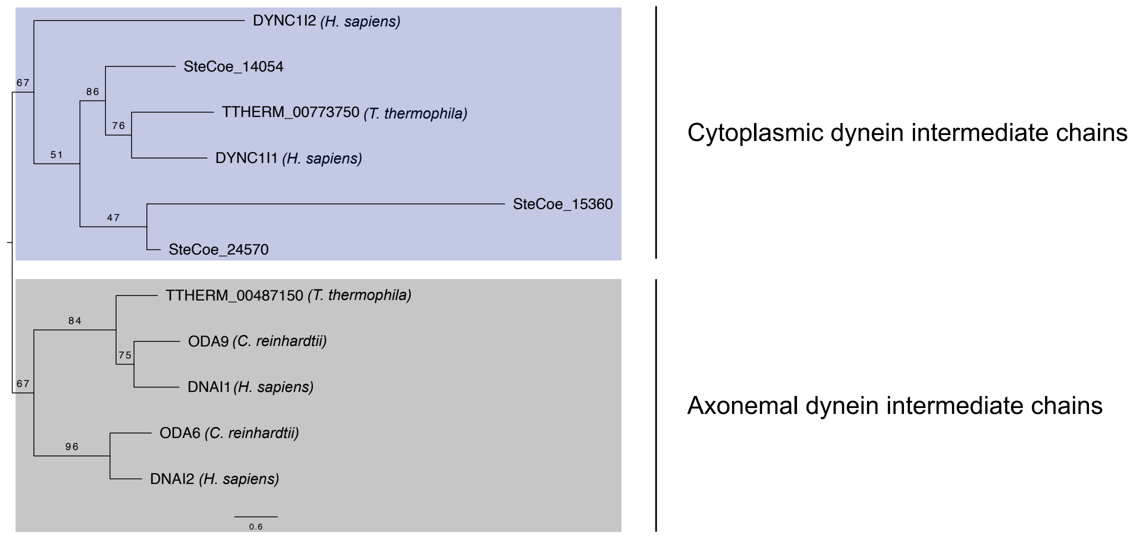

Supplementary Figure 3. Phylogenetic tree of dynein intermediate chains. Maximum likelihood phylogeny of dynein intermediate chains from *Homo sapiens*, *Tetrahymena thermophila*, *Chlamydomonas reinhardtii*, and the three *Stentor coeruleus* dyneins observed here. Tree was constructed using PhyML with 100 bootstrap replicates. Node labels indicate bootstrap support values. Scale bar represents substitutions per site. Cytoplasmic dynein intermediate chains are highlighted in blue, axonemal dynein intermediate chains are highlighted in gray.

Supplementary Files

Supplementary Table 1. Kallisto raw data table – bulk AP RNA sequencing

Supplementary Table 2. Batch corrected counts – bulk AP RNA sequencing

Supplementary Table 3. Differential enrichment analysis with PyDeSeq2 – bulk AP RNA sequencing

Supplementary Table 4. Domain enrichment analysis - Anterior

Supplementary Table 5. Domain enrichment analysis - Posterior

Supplementary Table 6. Kallisto log normalized tpm table – tubulin half-cell RNA sequencing

Supplementary Table 7. Skew analysis – tubulin half-cell RNA sequencing

Supplementary Table 8. Kallisto log normalized tpm table – dynein half-cell RNA sequencing

Supplementary Table 9. Skew analysis – dynein (G04) half-cell RNA sequencing

Supplementary Table 10. Skew analysis – dynein (G05) half-cell RNA sequencing
